# Hippocampal output profoundly impacts the interpretation of tactile input patterns in SI cortical neurons

**DOI:** 10.1016/j.isci.2023.106885

**Published:** 2023-05-13

**Authors:** Leila Etemadi, Jonas M.D. Enander, Henrik Jörntell

**Affiliations:** 1 Neural Basis of Sensorimotor Control, Department of Experimental Medical Science, Lund University, Lund, Sweden; 2 Center for Social and Affective Neuroscience, Linköping University, Linköping, Sweden

**Keywords:** Sensory neuroscience, Cognitive neuroscience

## Abstract

Due to continuous state variations in neocortical circuits, individual somatosensory cortex (SI) neurons *in vivo* display a variety of intracellular responses to the exact same spatiotemporal tactile input pattern. To manipulate the internal cortical state, we here used brief electrical stimulation of the output region of the hippocampus, which preceded the delivery of specific tactile afferent input patterns to digit 2 of the anesthetized rat. We find that hippocampal output had a diversified, remarkably strong impact on the intracellular response types displayed by each neuron in the primary SI to each given tactile input pattern. Qualitatively, this impact was comparable to that previously described for cortical output, which was surprising given the widely assumed specific roles of the hippocampus, such as in cortical memory formation. The findings show that hippocampal output can profoundly impact the state-dependent interpretation of tactile inputs and hence influence perception, potentially with affective and semantic components.

## Highlights

• Tactile input patterns drive SI neurons via multiple dynamically gated pathways
• Hippocampal output differentially gates the transmission in these pathways
• Hippocampal output is thereby part of cortical anticipation, equivalent to a “prior”
• Its function may be to impact semantic and emotional aspects in tactile perception

## Graphical abstract

**Figure:**
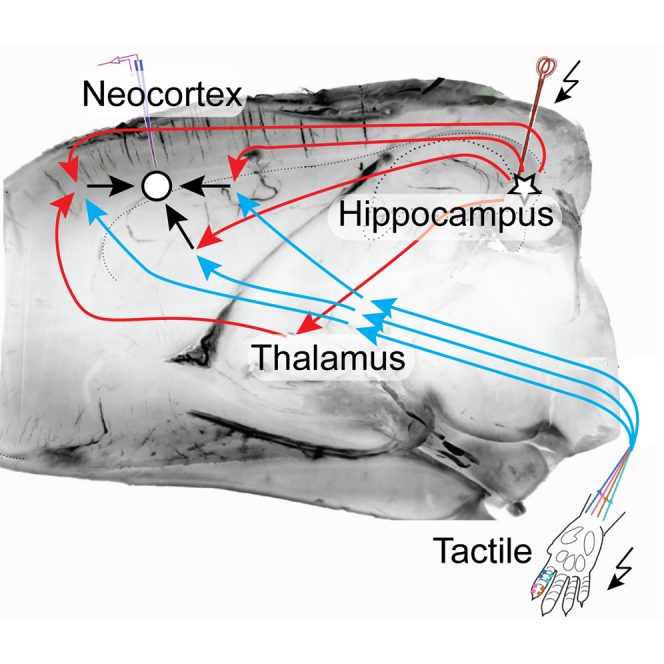

## Introduction

Analysis of the human brain has indicated that the neocortex is functionally a heavily interconnected network.^1^ Various analyses in the rodent tell a similar story.^2^^,^^3^^,^^4^ We have previously shown that the processing of tactile information also at the cellular level in the primary somatosensory cortex (SI) is dependent on other parts of the cortex. This was shown both by stroke-like lesions and by electrical manipulation of brain activity in neocortical areas with a remote location to the SI.^5^^,^^6^ Recent anatomical studies have indicated that the extent of connections made by individual corticocortical axons have previously been greatly underestimated^7^^,^^8^ (see also https://www.janelia.org/project-team/mouselight; https://mouse.braindatacenter.cn/), hence giving structural support for the observations that information in the neocortex is globally integrated. Given that perception to some extent depends on expectation, which through the internal cortical state may exert impact already before the tactile afferent information enters the SI cortex, these findings suggest that tactile perception could be influenced by a globally integrated action across the neocortex.

The hippocampal formation is closely interconnected with the neocortex. The hippocampus receives sensory information from a large number of different neocortical areas feeding, via the entorhinal cortex, into the subiculum.^9^ Indeed, the hippocampus is also engaged in processing of tactile input.^10^ Through its output via the subiculum, the hippocampus can monosynaptically activate the anterior thalamus^11^ and indirectly thereby also widespread areas of the cortex. Subiculum neurons can also have widespread projections, such as to the hypothalamus, regions of the temporal/entorhinal cortices surrounding the hippocampus, and the ventral striatum,^11^^,^^12^ which also enables the hippocampus to potentially impact the activity across widespread areas of the neocortex.

Using intracellular recordings from SI neurons, we have previously shown that repeated presentations of a given spatiotemporal tactile input pattern from digit 2 combine with the state of the neocortical network to result in a variety of response states in SI neurons.^13^ This finding indicated that traditional analysis methods involving response averaging would risk missing essential aspects of how the neocortical neurons process information, related to the perceptual process. Interestingly, the relative orthogonality of these response states suggested the presence of attractor-type dynamics,^13^^,^^14^ reminiscent of what has also been proposed to characterize the neuron population activity dynamics in the hippocampus.^15^^,^^16^ More recently, we showed that manipulation of the internal cortical state through electrical activation of a remote neocortical area can change the preferred network solutions, or the types of response states observed in SI neurons, for every given tactile input pattern.^5^ Remarkably, this change occurred despite that the remote cortical perturbation did not induce any overt response in the recorded neurons. That finding suggested that the effects on the response states in individual neurons were indirectly mediated via more subtle changes in the state of the neocortex globally, which in turn would alter the number of “open” network pathways supplying the recorded neuron with information related to the tactile event as a function of time (i.e., cortical state). Such gating of input pathways and information distribution across the neocortex is likely part of the central mechanisms for generating perception, and alteration of such pathways could be a root cause of illusions. Here, we address the question if also the hippocampal output, generated by electrical activation of its neural output stage in the subiculum, can impact the responses in SI neurons to given spatiotemporal tactile input patterns. We find that the hippocampal activation dramatically altered the responses of the SI neurons to tactile inputs, suggesting that the state of hippocampal activity could have a profound effect on the cortical interpretation, or perception, of such inputs. Perhaps the most surprising aspect is that we found no major qualitative difference between the present hippocampal impact versus the previously explored remote cortical impact,^5^ despite that the hippocampus is widely considered to have very different specific roles in neocortical processing and memory.

## Results

**Figure 1:**
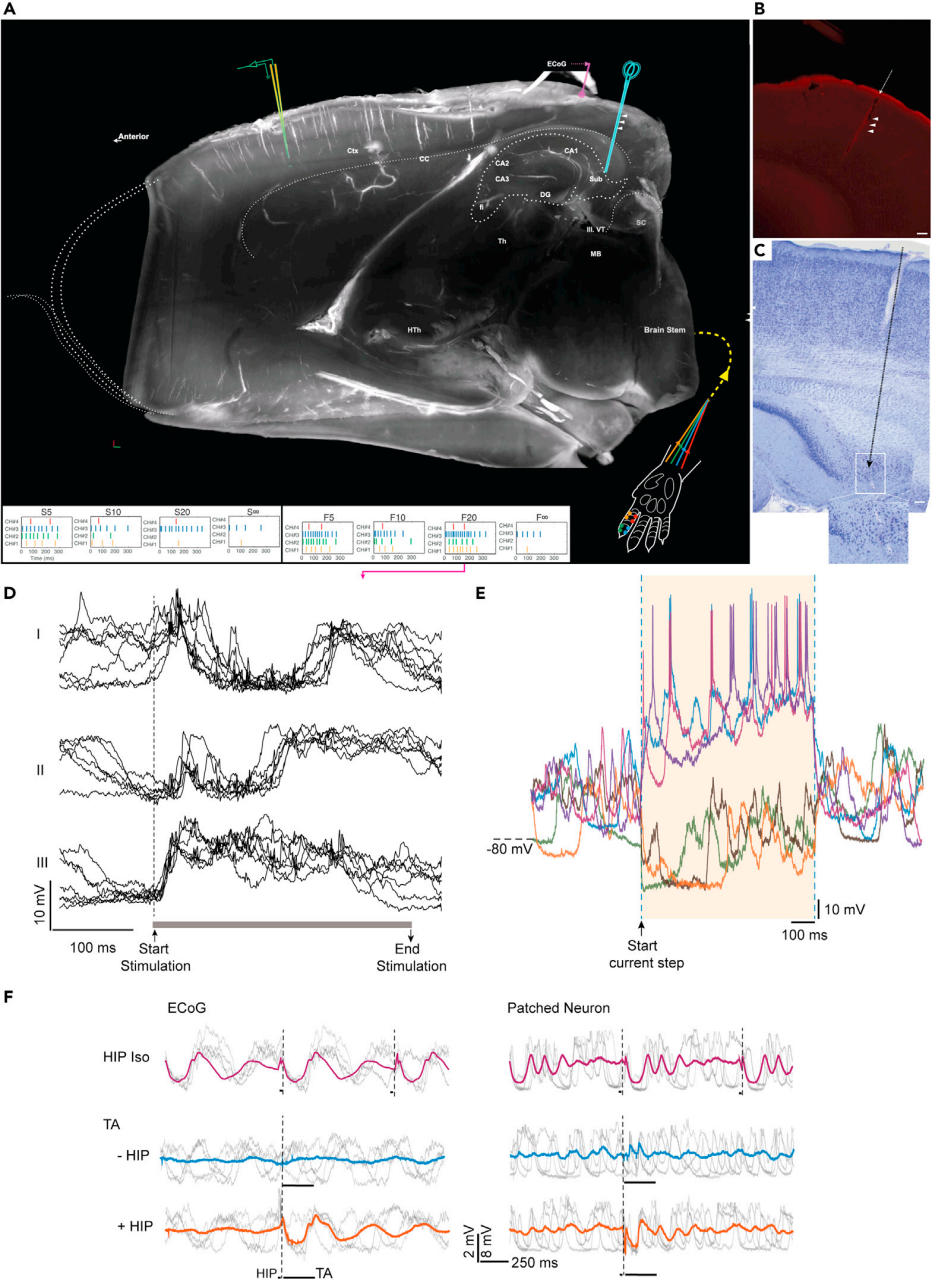
Experimental setup (A) Location of the stimulation and recording electrodes visualized in one hemisphere clarified via the iDISCO Clearing protocol and visualized using light-sheet microscopy. White arrowheads indicate identified location of the HIP stimulation electrode (blue cartoon electrode, with the tip located in the subiculum/terminal CA1 region). In the bottom part of this image, the second digit of the left forepaw along with the location of four pairs of electrodes (color coded, corresponding to different tactile “channels”, “CH”) are shown together with the eight different spatiotemporal patterns of tactile stimulation (TA). The patch-clamp recording (indicated by a yellow/green cartoon electrode) was made in the cortical SI area. (B) HIP stimulation electrode track identified within the clarified brain tissue (white arrowheads). The stimulation electrode was covered in neurobiotin solution before the experiment. The presented image is a snapshot captured by light-sheet microscopy following conjugating neurobiotin with the streptavidin Alexa Flour-568 (red color in the image). Scale bar: 0.1 mm. (C) Histological identification of an HIP stimulation electrode track in regular light microscopy using cresyl violet acetate staining technique. The location of the tip of the HIP electrode in the subiculum area is visible in the white square area as a slight “burn” mark, shown also with further magnification below. Scale bar: 0.1 mm. (D) Examples of intracellular response types (I-III) evoked by repeated stimulations using the tactile input pattern F20. The dashed line indicates the starting point, and the gray line indicates the duration of the stimulation. Some remnants of the blanked shock artifacts created minor spikes in the recording. (E) Intracellular responses evoked by current step commands (+0.6 nA and −0.6 nA, respectively) used for controlling the recording quality. (F) Electrocorticogram (ECoG) (left) and intracellular responses (right) to the isolated subiculum stimulation (HIP iso), the TA input pattern F∞ without and with preceding HIP stimulation (HIPTA). Thick traces in each subpanel represent averages. Gray thin traces are example of raw data traces. Dashed vertical lines indicate the onsets of the stimulations (which occurred at a higher frequency for the HIP iso condition), which in the case of the HIPTA condition (bottom) consisted of the briefer HIP stimulation preceding the longer TA stimulation (as indicated at the bottom).

We made whole-cell patch-clamp recordings from putative pyramidal neurons (see STAR Methods; also^13^) between layers III-V in the forepaw region of the SI cortex, while stimulating the distal part of the second digit with eight fixed spatiotemporal tactile activation (TA) patterns delivered in pseudo-random order (Figure 1A), a protocol we have used in numerous previous publications.^5^^,^^6^^,^^13^^,^^17^^,^^18^^,^^19^ Here, these TA input patterns were provided in isolation or in combination with a preceding brief stimulation of the subiculum/terminal CA1 region of the hippocampus (HIPTA) (Figures 1A–1C). As the study relied on comparisons between individual raw responses with large variance, we needed a high number of repetitions of each stimulus, and we could therefore include only recordings which maintained a high quality for a duration of at least 25 min (N = 9 neurons).

As we have reported earlier,^5^^,^^13^ each individual neuron in SI cortex displayed a wide variety of response types when the exact same TA stimulus was repeatedly presented (Figure 1D). This indicates that response averaging would risk missing essential features of these cortical responses as averaging would tend to overemphasize the impact of the peripheral component of the response versus its internally generated components. Since the latter presumably is essential to the perceptual process in the cortex, which we were interested in here, we instead focused on the characterization of the pool of individual raw responses evoked by each stimulus pattern and each stimulus condition. This process involved the step to cluster the responses evoked by the same stimulus pattern. We used a clustering method based on the temporal profile of the evoked responses (Figure 1D; see also^5^), where the temporal response profile reflects the temporal structure of the responses in the different subnetworks forwarding input to the recorded neuron.^5^ Clustering relied on analysis of spike-free intracellular responses, which reflect the synaptic input to the cell. In each recording we therefore applied a mild hyperpolarization current (in the order of −10 pA) to prevent the neuron from spiking (the occasional few remaining spikes could be blanked in software), whereas the quality of the recording was occasionally verified by injecting depolarizing current to evoke spike responses (Figure 1E).

Stimulation of the subiculum (HIP) in isolation (iso) typically evoked detectable responses in the average ECoG signal (recorded from the parietal cortex, Figure 1A), which were several order of magnitudes longer than the duration of the stimulation (7 pulses at 333 Hz, i.e., 18 ms), whereas the TA stimulation alone evoked no detectable response in the ECoG (Figure 1F, left column). In the average intracellular signal of the recorded SI neurons, this HIP response was often much less distinct and typically less long lasting than in the ECoG, although it did induce a clear tendency toward oscillation in the intracellular signal (Figure 1F, right column). In contrast, the average response to the TA stimulus was always clear in the intracellular recordings from the SI neurons (though widely different from one neuron to the other, see^13^). Notably, the intracellular responses evoked by HIP iso and by the TA pattern were clearly different from the HIPTA response, indicating that the effect on the global cortical state induced by the HIP stimulation was nonlinearly combined with the impact of the TA input to produce a new response. Figure S1 presents a frequency distribution analysis of the intracellular signal across the neurons, which indicated that the HIP stimulation pushed the intracellular signal in the low frequency range to a somewhat higher frequency, i.e., in line with the observation of the oscillatory response components induced by the HIP stimulation in Figure 1F.

**Figure 2:**
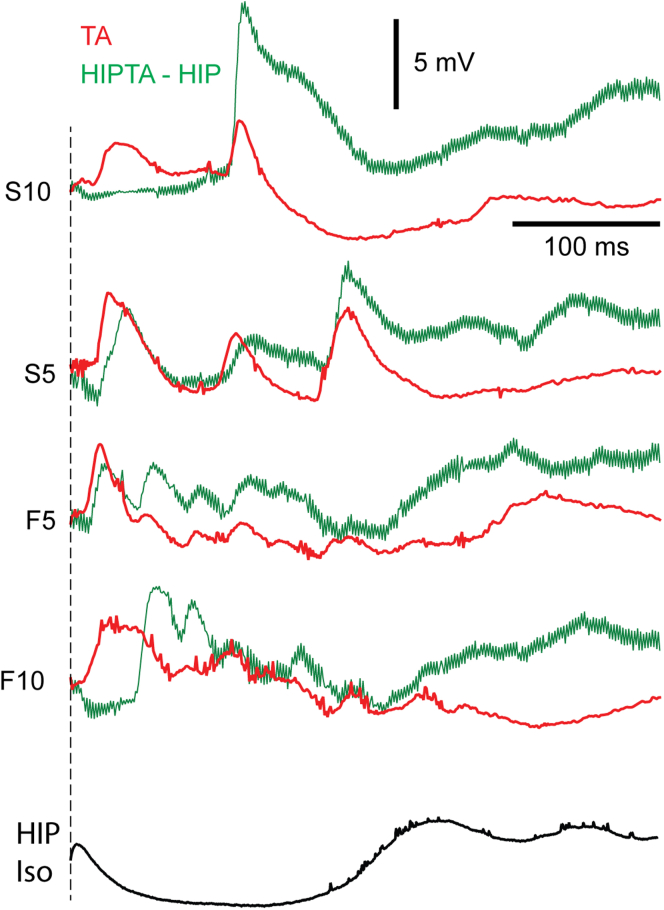
Impact of hippocampal stimulation (HIP) on average responses evoked by specific TA patterns The red traces illustrate the average intracellular response to the indicated TA input. The green traces are the corresponding average traces evoked by the same TA inputs when they were combined with the HIP stimulation (HIPTA), where the average response to the isolated HIP stimulation was subtracted from the HIPTA responses to illustrate only the TA component of the HIPTA responses (HIPTA-HIP). The bottom trace is the average response to the HIP Iso stimulation.

Interestingly, the combination of a TA input with the preceding HIP stimulation could generate widely different outcomes across different TA input patterns. Figure 2 illustrates examples of the impacts the HIP stimulation could have on the average responses evoked by four different TA patterns in the same cell. Notably, the hippocampal output caused the S10 TA input pattern to completely lose an early excitatory component, whereas a later excitatory component was instead greatly amplified. For the S5 TA input pattern, the early excitatory component was in contrast not eliminated, in fact a bit amplified but also slightly slowed down. Also the impacts on the two other example TA input patterns F5 and F10 were highly different. These results indicate that the TA and the HIP inputs combined almost exclusively nonlinearly. Nonlinear combinations could for example be explained by the fact that multiple intracortical pathways that mediate the TA synaptic input to the recorded neuron were differentially nonlinearly controlled by the given HIP input, even at different time points during a response. The differential effect across the TA input patterns would then moreover be mediated by partly different such pathways as the same HIP input could have widely different impact at the same time point, depending on the TA input.

### Profound impact of the HIP stimulation on the TA response types

**Figure 3:**
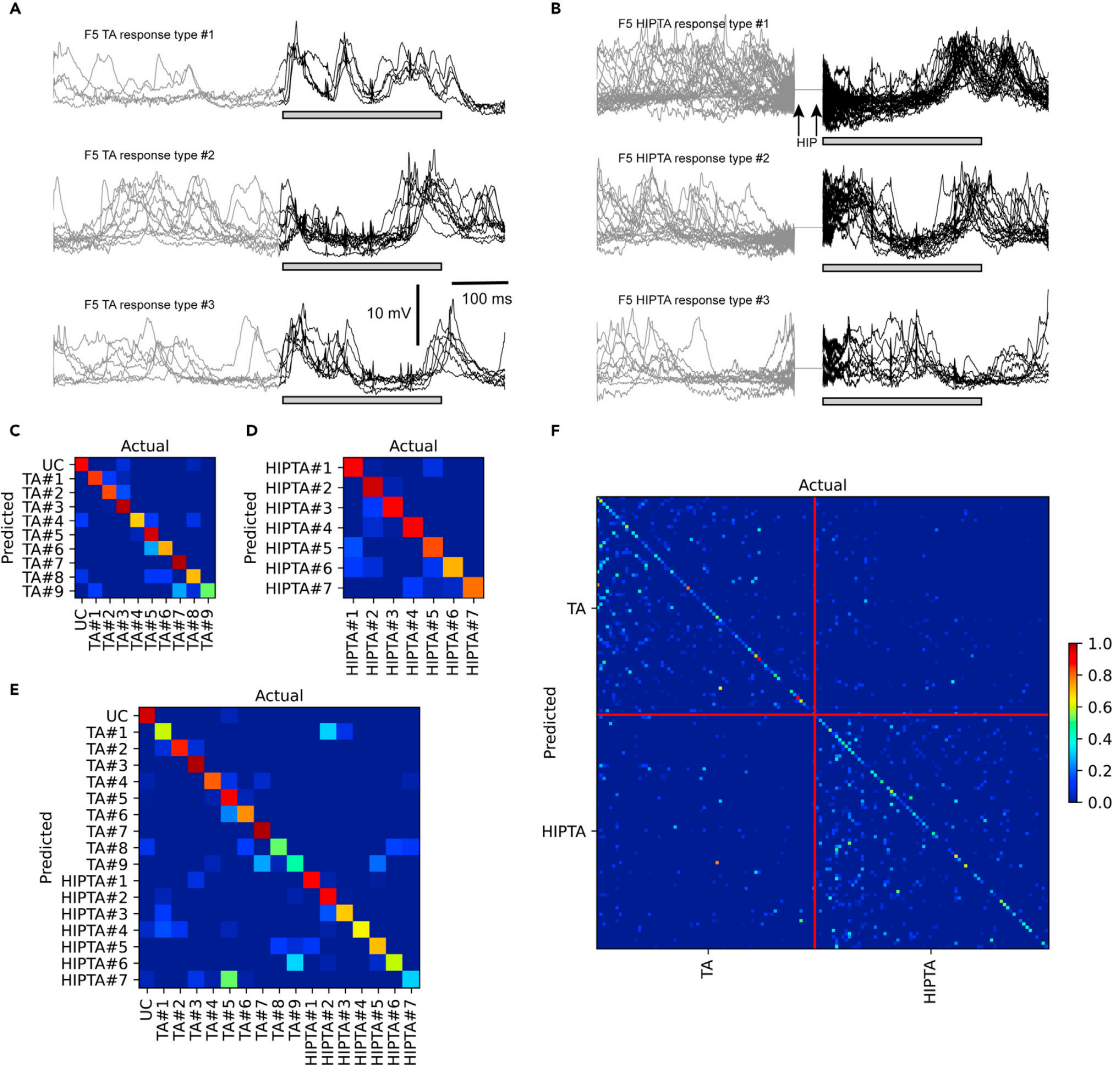
The intracellular response types evoked by a given tactile input pattern (TA) were profoundly altered when combined with subiculum stimulation (HIPTA) (A) Examples of superimposed raw intracellular responses for three response types evoked by repeated activation of one specific tactile input pattern (F5). Black part of the traces represent the time period when there was ongoing TA input (the duration of the actual stimulation is indicated by the gray bars); the preceding gray part of the traces were recorded before the onset of the TA input pattern. (B) Response types evoked in the same cell and using the same tactile input pattern when preceded by the HIP stimulation. The HIP stimulation occurred during the blanked period of the recording. Note that some blanked shock artifacts of the TA input pattern left some remnants, which almost looked like small spikes in the intracellular signals. (C) Confusion matrix for the nine response types evoked by the F5 tactile input pattern, following a mapping of these response types to principal component space. The presence of a “non-blue” diagonal in the matrix indicates that the identified response types, evoked by one and the same stimulation pattern, were highly distinct from each other (as previously analyzed in detail^5^^,^^13^). UC, unclassified responses. (D) Similar display for the seven identified response types when the F5 tactile input pattern was preceded by hippocampal output. (E) Confusion matrix for the response types evoked by the F5 pattern under the two conditions (TA and HIPTA, without and with preceding hippocampal output). (F) Confusion matrix for the responses evoked by all eight tactile input patterns (Figure 1A) across the two conditions TA and HIPTA. Red vertical and horizontal lines divide the two conditions.

We next made a detailed analysis of the response types evoked in the SI neurons. Different intracellular response types evoked by the same TA input pattern are a striking phenomenon that we have previously reported.^5^^,^^13^ Response types identified among a set of individual raw responses can be demonstrated with response clustering, and here we explored if these response type clusters formed by the pool of raw responses evoked by a single TA input pattern were impacted by preceding HIP stimulation. Figure 3A illustrates some example response types evoked by the F5 TA input. Figure 3B presents responses to the same TA input pattern but now preceded by the brief HIP stimulation. In this case, the response types evoked by the TA input pattern were profoundly altered compared to Figure 3A. This can for example be seen in that response peaks were eliminated or added at different points in time during the duration of the TA input pattern when it was combined with preceding HIP stimulation. In agreement with what we have previously reported,^5^^,^^13^ Figures 3A and 3B show that the differences between the individual intracellular responses evoked by the exact same TA input were very large, to an extent that would imply that response averaging would miss the dominant aspects of the information present in these recordings. Nevertheless, in traditional quantitative terms, all of the 9 neurons recorded from here did display a substantial average EPSP response (>3 mV, peaking no later than 20 ms after the onset of the TA stimulation) for at least one of the TA patterns (Figures 1A and 2). As we have analyzed in great detail before,^13^^,^^19^ the different SI neurons displayed different average responses, and response types, to the same given TA inputs (not illustrated).

In order to verify that the differences in response types observed in the above example occurred systematically, we performed a quantitative comparison of the temporal profiles of the responses using a principal-component analysis (PCA)+kNN (k-Nearest Neighbor) classification analysis (see Methods; same approach as that used by Etemadi et al.^5^). We first compared the clusters evoked by the same TA input pattern but separated based on the condition (i.e., with or without preceding HIP stimulation); hence, in Figure 3 the TA and the HIPTA response types were considered different sets and therefore clustered independently of each other. This analysis showed that the nine clusters of response types evoked by this TA input pattern were systematically distinct, with little confusion (similarity) between the response types (Figure 3C). This was also true for the seven clusters evoked by the F5 HIPTA input condition (Figure 3D). But the more critical comparison was that between the TA and the HIPTA clusters. Figure 3E shows that all response types, except the TA#5 and the HIPTA#7 response clusters, were well separated from each other.

Whereas the preceding panels only illustrated responses evoked by the F5 example TA input, Figure 3F illustrates that, even when all response clusters for all eight stimulation patterns, across the two input conditions, were compared, the confusion between all of these response clusters was still highly limited. These results hence indicate that the HIP conditioning stimulus profoundly impacted the response types evoked by each given TA input and also confirmed our previous findings that the different response types evoked by each TA pattern is relatively unique compared to the response types evoked by other TA input patterns.^13^ Figure 3F also indicates that the HIP condition did not in this respect alter the relationship between the response types evoked by the different TA input patterns, i.e., they were still a relatively unique set for each TA input. This is interesting as it implies that whereas each of the eight TA input patterns typically evoked around 7 response types (as can be read out from Table 1 indicating that the chance level per each pattern was about 15%), under the HIP condition there were still about 7 response types per pattern, but the whole set of response types were also different from those evoked by the corresponding TA input without the HIP condition. This suggests that the HIP stimulation resulted in a whole new set of network solutions to each individual TA input pattern.

**Table 1:** Separability of the clusters for each clustering and classification context as measured by the mean F1 score

Figure	Context	Mean F1 (±s.d.)	Mean chance level (±s.d.)	N (cells)
3C	F5	83% (±7%)	14% (±3%)	9
–	S5	82% (±5%)	17% (±4%)	9
–	F10	83% (±6%)	15% (±3%)	9
–	S10	80% (±6%)	15% (±3%)	9
–	F20	83% (±7%)	15% (±4%)	9
–	S20	80% (±4%)	14% (±4%)	9
–	F∞	83% (±4%)	15% (±7%)	9
–	S∞	83% (±7%)	17% (±8%)	9
3D	F5 HIP	82% (±7%)	16% (±3%)	9
–	S5 HIP	80% (±7%)	14% (±3%)	9
–	F10 HIP	79% (±6%)	15% (±5%)	9
–	S10 HIP	83% (±7%)	16% (±5%)	9
–	F20 HIP	83% (±7%)	15% (±3%)	9
–	S20 HIP	79% (±8%)	16% (±5%)	9
–	F∞ HIP	82% (±8%)	15% (±2%)	9
–	S∞ HIP	81% (±7%)	17% (±4%)	9
3E	F5 pooled	66% (±15%)	7% (±1%)	9
3E	S5 pooled	67% (±14%)	8% (±2%)	9
3E	F10 pooled	66% (±12%)	7% (±2%)	9
3E	S10 pooled	69% (±8%)	8% (±2%)	9
3E	F20 pooled	68% (±11%)	7% (±1%)	9
3E	S20 pooled	66% (±11%)	7% (±2%)	9
3E	F∞ pooled	65% (±12%)	7% (±2%)	9
3E	S∞ pooled	68% (±10%)	8% (±2%)	9
3F	Cell	23% (±11%)	0.9% (±0.1%)	9

The data in this table are the mean and standard deviation from the entire population of recorded cells. Note that the chance level depends on the number of clusters and varies for each neuron and pattern and cell and that all F1 scores were many multiples above chance level. The HIP suffix refers to TA patterns preceded by HIP stimulation. The “Pooled” suffix refers to when response clusters from the TA and HIPTA conditions were compared together. The “Cell” context represents classification across all TA and HIPTA clusters for a neuron.

Across the population of neurons we recorded from, these quantitative measures were highly consistent (Table 1).

### Hippocampal conditioning generated unique response clusters in SI neurons

**Figure 4:**
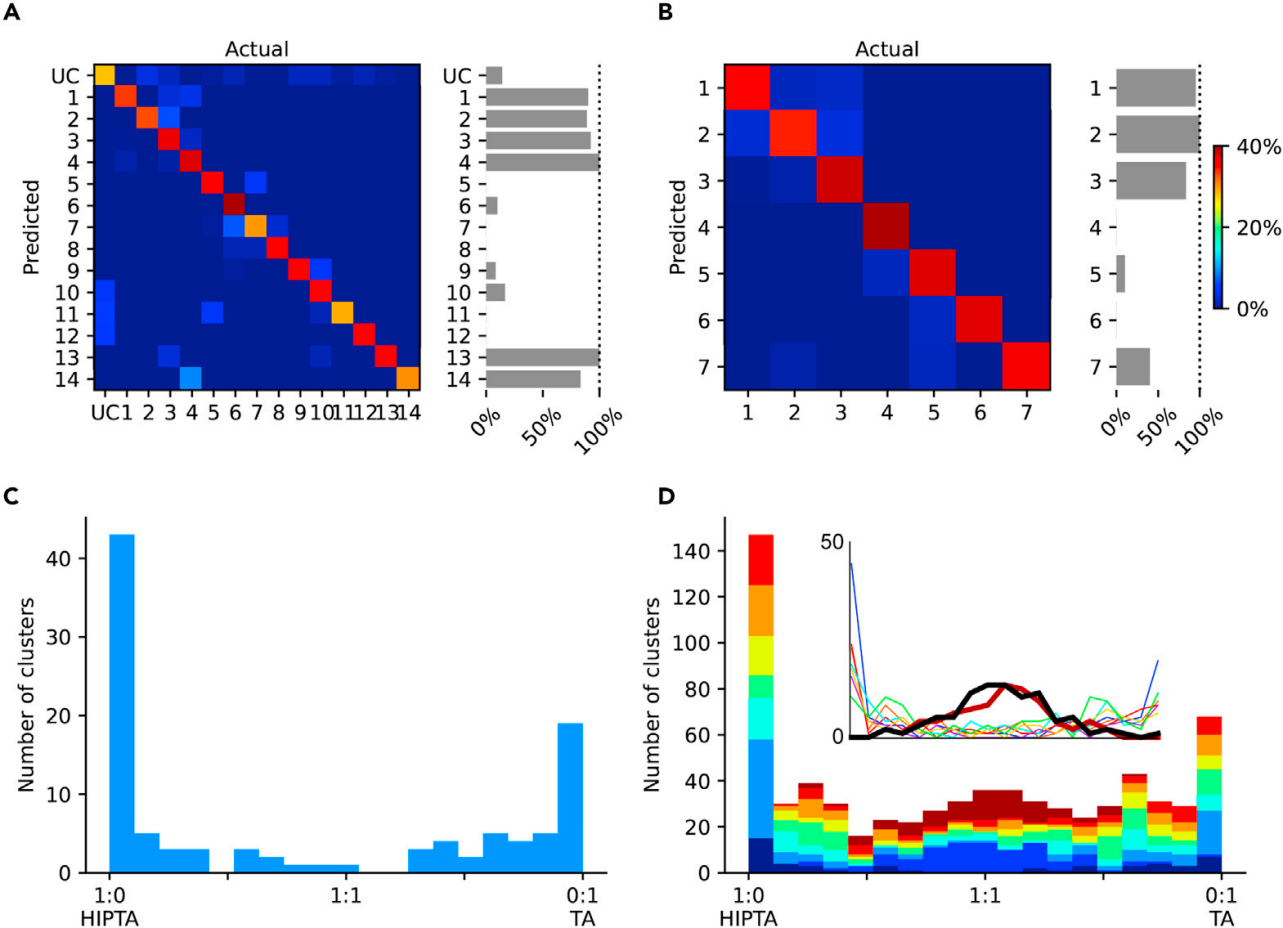
When the clustering algorithm was agnostic of the condition, identified response types were still relatively unique for each condition (A) Confusion matrix for all response types evoked by one tactile input pattern (F5, the TA and the HIPTA conditions pooled) and the percentage of responses evoked with preceding hippocampal output for each response type. UC, unclassified responses. (B) Similar display for the responses evoked by another tactile input pattern (S20). (C) For each response type identified in this cell, across all TA input patterns, the proportion of the two conditions under which they were evoked. (D) Similar display as in (C) but for all cells recorded. Each cell was represented by a unique color. Inset represents each cell individually by a colored line, where the two cells without any clear sorting between stimulus condition and response type are indicated by thicker lines.

Whereas the analysis in Figure 3 assumed that the TA and the HIPTA response types were different sets and therefore clustered independently of each other, Figure 4 instead assumes that TA and HIPTA responses were part of the same distribution. Therefore, in this case the clusters were identified without an *a priori* separation of the responses based on condition. Importantly, the clustering algorithm could still identify clusters which with a very high probability consisted of responses of only one or the other condition. This is illustrated for one example neuron, for two different TA input patterns, F5 and S20, in Figures 4A and 4B. Across all eight TA input patterns, for the same example neuron, this was a consistent finding (Figure 4C). In our population of recorded neurons (N = 9), almost all tended to a bimodal distribution (Figure 4D), whereas only two of the neurons had a more Gaussian-like distribution (for example, the neuron represented by brown bars in Figure 4D). In the inset, each neuron is represented individually, and these two outlier neurons are indicated by thicker lines (they were outliers also in the frequency analysis presented in Figure S1). The other 7 neurons had very little confusion between the two conditions, i.e., they had strong bimodal distributions. Overall, these findings underscore that the HIP conditioning stimulation profoundly impacted the response types evoked by each given TA input since the clusters tended to contain responses from only one or the other condition.

## Discussion

Here we showed that brief preceding electrical activation of the subiculum, an input-output hub of the hippocampus, profoundly impacted the responses to tactile input patterns in SI neurons, located a long distance from the subiculum/CA1 terminal region (Figure 1A). As we have previously described,^5^^,^^13^ the variety of intracellular response states evoked by each individual tactile input pattern indicates that there is a range of dynamically gated cortical subnetworks supplying the sensory input to each neuron. The cortical network can thereby be described as containing a wide range of network solutions for any given spatiotemporal pattern of sensory input. What we described here is that the specific cortical network solution that applies at a given moment also depends on hippocampal activity. Hence, as further detailed below, hippocampus is part of what defines the potential circuitry mechanisms underlying perception by impacting the predictive or anticipative code in the neocortical circuitry.

Though the relative physical distance between SI and subiculum is large, neurophysiologically the distance is not so large. The subiculum projects monosynaptically to the anterior thalamus. From the thalamus, the subiculum output may reach the SI cortex within just a few synapses. There are also other potential pathways, for example, to hypothalamus and ventral striatum and then to thalamus, which is another potential route by which hippocampal output can impact SI activity indirectly. Even so, the magnitude of the impact that we observed here was surprising. The synchronized activation that was achieved by the hippocampal microstimulation likely made the effect more powerful than a normally generated hippocampal output. On the other hand, the observed effects remained for several 100 ms after the termination of the activation of the output. In addition, a previous study showed that there can be a strong correlation between SI neurons in deep cortical layers and hippocampal neurons during oscillatory activity.^20^ And, hippocampal neurons can be active at high firing rates,^21^^,^^22^ not seldom in relative synchrony.^23^ This suggests that hippocampal output can continually modify the global cortical state with high potency, including impacting the responses of SI neurons to tactile stimuli.

The hippocampus is by many authors considered to be at the “top” of the hierarchy of cortical processing, and it is considered critical for memory consolidation, i.e., to drive synaptic plasticity in the neocortex via replay of previous experiences.^24^ In comparison with our previous study of the perturbation of SI tactile processing generated from a remote cortical area, one would thus expect quite different effects from the hippocampal output, for example, qualitatively different impacts on the intracellular signal in the SI neurons. However, in short, the effects we found were instead remarkably comparable to those we obtained from a remote location in the neocortex.^5^ In comparison, the hippocampal output seemed potentially more potent in generating long-lasting oscillatory activity (Figures 1 and S1), possibly through a more efficient synchronization of the global thalamic activity, in turn possibly due to the impact of the hippocampus on the anterior thalamus.^11^ Otherwise, we observed qualitatively similar nonlinear effects, i.e., that the HIPTA responses were strongly nonlinear combinations of the TA and the HIP responses. Such nonlinear effects are in turn indicative of that the perturbation impacted the gating of transmission of tactile information from wide areas of the neocortex to the SI neurons.^5^ But the fact that the impact of hippocampal output here did not qualitatively differ from neocortical output raises the interesting possibility that hippocampal cortex may not exert such a different neocortical impact compared to neocortex itself, in neurophysiological terms, in the time window of 1 h.

Our low-noise intracellular recordings represented the temporal evolution of the combined activity of thousands of neurons being afferent to the recorded neuron,^25^ and the specific trajectories observed reflect how those thousands of inputs are differentially gated. As we have previously underscored, the fact that the cluster analysis identified multiple preferred response states does not imply that these response states are fixed entities or the only response states possible.^5^^,^^13^ The cluster analysis merely indicates that there are some central response features that some responses share, whereas at least one of these features was not shared by the members of the other clusters. The unique, in fact, the orthogonal, nature of these response states (Figure 3; Table 1) indicates that these features may correspond to multiple independent network solutions, which are dynamically gated as a product of the cortical internal state and how it combines with the external sensory input. The relative orthogonality between these response states, as indicated by the PCA/decoding analysis, indicates that the network in this regard may work according to attractor dynamics,^5^^,^^13^ conceptually introduced in a recent review.^26^

### Broader implications of our findings

By focusing on the trial-to-trial response variability, rather than different forms of response averaging, which is otherwise a very common neurophysiological approach, our approach takes the alternative angle of addressing how the hippocampal-driven cortical state would impact the SI neuron responses to given sensory input patterns. Hence, we move beyond the idea of considering the neocortex as essentially driven by external stimuli but instead focus on what degree of response variation can be generated as a consequence of the cortical state. This approach is in agreement with recent perspectives arguing that the paradigm of a sensory-driven cortex is by itself inadequate and that we need to address how changes in activity intrinsic to the brain impact the representation of sensory input,^27^ which is in turn considered needed to explain cognition. This approach is well in line with recent neuroanatomical studies indicating that the profusion of global interconnections across the neocortical neuronal networks has been vastly underestimated^7^ (https://www.janelia.org/project-team/mouselight; https://mouse.braindatacenter.cn/) and that also the cortico-thalamo-cortical pathways are much more widespread^28^ than what is typically accounted for in models of cortical network operation. Such profuse connectivity would seem to inevitably lead to widespread representations of any cortical signals, including those driven by sensory inputs. Indeed, several recent analyses at the cellular level indicate that the cortical representation of tactile information is dependent on globally distributed networks.^6^^,^^17^^,^^18^^,^^29^^,^^30^ Hence, these earlier findings speak in favor of the cortex being a highly recurrent network, rather than having a predominantly feedforward network architecture. Here we show that this recurrent network seems to include even the hippocampus.

In a recurrent cortical neural network architecture, it is to be expected that a tactile input pattern, or in principle any other sensory input pattern, would combine with the ever-evolving state of the neocortical network. This state would equal a component of the ‘‘prior,’’ ‘‘expectation,’’ or ‘‘prediction’’ currently residing in the neocortical network. That internal state component will be an important determinant of the resulting activity distribution across all neurons of the cortical circuitry that results when the tactile input pattern arrives—which is a potential definition of a ‘‘percept.’’ Our results indicate that the diversity of cortical response states is profoundly impacted by hippocampal output. Whatever information that is being processed in the hippocampus will hence to some extent impact how we perceive a given tactile input, which for example could contribute to semantic and emotional aspects in haptic perception.^31^^,^^32^^,^^33^

### Limitations of the study

As we have extensively analyzed and discussed elsewhere,^5^^,^^13^ the anesthesia inevitably impacted the recorded responses but not to an extent that would invalidate the main finding that hippocampal output shifted the global cortical state to an extent that it would alter the SI neuron representations, at the integrated level translating to the percept, of given tactile input patterns. Furthermore, the physiological network structure, quantified as the recruitment order of a high number of simultaneously recorded cortical neurons across different sources of network excitation, does not alter with the type of anesthesia used here.^34^^,^^35^ Also, the type of network attractor dynamics that we observed here is likely closely related to the type of neuron population-level dynamics observed in the hippocampus,^15^ which remains stable whether the animal is awake or in deep sleep.^16^ Notably, using the same anesthesia as we have used here, highly detailed features of visual cortical processing have been discovered.^36^^,^^37^

## STAR★Methods

### Key resources table

**Table:** 

REAGENT or RESOURCE	SOURCE	IDENTIFIER
**Deposited data**		

Recording data	This study	https://doi.org/10.6084/m9.figshare.22758686.v1

**Experimental models: Organisms/strains**		

Sprague-Dawley WT	Taconic	N/A

**Software and algorithms**		

Spike2	CED	ced.co.uk
MATLAB	MathWork	https://www.mathworks.com/
Python	Python	https://www.python.org/

### Resource availability

#### Lead contact

For any additional inquiry and information related to materials and resources used in this work Henrik Jörntell is the lead contact (Henrik.Jorntell@med.lu.se).

#### Materials availability

This study did not use any new/unique materials and/reagents.

### Experimental model and study participant details

We made recordings from 9 anesthetized male adult Sprague-Dawley rats (weight: 250–380 g, age: 12 ± 2 weeks) in acute preparations. Before experiments, animals were maintained by Lund University animal facilities with 12h light/dark condition, 2–3 animals per cage (type 3H), and with free access to food and water.

#### Institutional permission

Ethical approval for this research was obtained in advance from the local animal ethics committee in Lund/Malmö (Ethical permit ID: M13193-2017).

### Method details

#### Surgical procedures

In order to make acute *in vivo* recordings, adult Sprague-Dawley rats were initially prepared in the same way as in previous studies.^5^^,^^13^ Briefly: 1) The animal was sedated by inhaling air mixed with isoflurane gas (3%, for 2 min); 2) To induce general anesthesia, a mixture of ketamine/xylazine (ketamine: 40 mg/kg and xylazine: 4 mg/kg, accordingly) was injected intraperitoneally; 3) an incision in the inguinal area of the hindlimb was made to insert a catheter in the femoral vein for continuous infusion of Ringer acetate and glucose mixed with anesthetic (ketamine and xylazine in a 20:1 ratio, delivered at a rate of 5 mg/kg/h ketamine). 4) A hemicraniectomy (∼2 × 2 mm) was made on the right side of the skull at the area covering the somatosensory cortex (SI) on the right-hand side (∼2 × 2 mm), located at (from bregma): Ap: - 1.0 - +0.1, ML: 3.0–5.0.

A second cortical exposure over the visual cortex was made to gain access to the caudal CA1 and the subiculum complex of the hippocampus, located at (from bregma): AP: −6, ML: 3. The stimulation electrode was advanced 5.8 mm at an angle of 8–10° to reach the subiculum complex. For the entire experiment, the EcoG was recorded via a surface electrode placed in the second cortical exposed area. To prevent tissue dehydration, the second exposed cortical area was covered with liquid paraffin oil. To stabilize the tissue and to prevent dehydration, the first cortical exposure was covered in agarose.

The anesthetics used were chosen because ketamine has previously been reported to not dramatically alter the neuronal recruitment order in spontaneous activity fluctuations and in stimulation-evoked responses as compared to the awake animal.^34^ As we have previously discussed extensively,^13^ anesthesia was required in order to achieve identical tactile stimulation patterns (where the electro tactile interface was the key, but would not be accepted in the awake animal) over a sufficiently long period of time. It also served to minimize brain activity noise caused by uncontrollable movements and internal thought processes unrelated to the stimuli. The level of anesthesia was assessed both by regularly verifying the absence of withdrawal reflexes to noxious pinch of the hind paw and by continuously monitoring the irregular presence of sleep spindles mixed with epochs of more desynchronized activity, a characteristic of sleep.^38^ The animal was sacrificed by an overdose of pentobarbital at the end of the experiment. For histological evaluations, the animals were perfused with 4% paraformaldehyde (PFA) and the brain was removed for further processing (see below).

#### Data collection*: In vivo* neuronal recordings

In order to analyze synaptic inputs, we performed whole cell (intracellular) recordings in the current clamp mode. Patch pipettes were made from borosilicate glass capillaries pulled to 6–10 MΩ of impedance, using a Sutter Instruments P-97 horizontal puller. The pipettes were back-filled with an electrolyte solution of (in mM): potassium gluconate (135), HEPES (10), KCl (6.0), Mg-ATP (2), EGTA (10). The solution was titrated to pH of 7.35–7.40 using 1 M KOH. Recording signals were amplified using the EPC-800 patch-clamp amplifier (HEKA Elektronik) in the current-clamp mode (bandwidth from DC up to 100 kHz). Throughout recording sessions, time continuous data was acquired and digitized at 100 kHz using the CED 1401 mk2 hardware controlled from the Spike2 software (Cambridge Electronic Devices, CED, Cambridge, UK).

The patch electrode was inserted into the SI area^39^ and advanced in a stepwise manner with applied positive pressure to prevent blockage of the electrode tip. The pipette was advanced in a slow forward movement (0.3 μm/sec) until a sudden change in the electrode resistance was detected. When a dramatic increase in the magnitude of the isolated spike, evoked or spontaneous, occurred, the positive pressure was switched to a brief negative pressure. Also, to create a more optimal condition for the establishment of a GigaOhm seal, a mild hyperpolarizing current (in the order of −10 pA) was applied to the electrode. Then, to gain access to the intracellular space, episodical negative pressures were applied to the electrode tip. The data recording started after obtaining low noise access to the intracellular environment with a stable signal. The recordings were characterized by stable membrane potential of < -55 mV in down states without any bias current, and with a peak-to-peak value between up and down states of >10 mV, with spike amplitudes of >25 mV in the beginning and end of stimulation protocols. During the stimulation protocols, the cells were prevented from firing with a mild hyperpolarizing current. All neurons recorded were putative pyramidal neurons rather than interneurons based on that they exhibited infrequent bursts of two or three spikes but had an absence of longer bursts or sustained periods of high firing.^35^ All recordings were made in the neocortical layers II/III-V (350–1100 μm of depth).

#### Design: Electrical tactile and cortical stimulations

Once an intracellular recording was obtained, the experiments consisted of delivering tactile stimulation patterns through an electro tactile interface, combined with hippocampal electrical activation. The electrotactile interface consisted of four bipolar pairs of percutaneous stainless steel needle electrodes (isolated except for the tip (0.2–0.5 mm) inserted into the superficial part of the skin of the second digit of the contralateral forepaw). Each pair represented a single channel for providing tactile stimulation (Figure 1A). Each electrode pair delivered a 400 μA constant current pulse with a duration of 200 μsec, which is about two times the threshold for activating tactile afferents using this type of stimulation,^40^ and below the threshold for activating nociceptive afferents.^41^

We used eight predefined spatiotemporal tactile afferent (TA) stimulation patterns (F5, S5, F10, S10, F20, S20, F∞, S∞; Figure 1A). These patterns were delivered to the skin in a preset randomized order as previously reported (Wahlbom et al.^6^^,^^13^^,^Enander et al.^17^^,^Oddo et al.^19^). The duration of each TA stimulation pattern varied somewhat but was less than 350 ms, and a randomized time interval of about 1.8 s separated the consecutive TA input patterns.^19^

Half of the TA inputs were combined with localized microstimulation in the subiculum/terminal part of the CA1 of the hippocampus. We used a glass-insulated tungsten electrode as previously described.^42^ In brief, the exposed tip of the electrode was about 100–150 μm to deliver the stimulation against a ground, consisting of a silver wire electrode inserted into the neck muscle. The microstimulation (HIP) consisted of cathodal constant current pulses of 0.2 ms duration and 0.4 mA intensity, delivered in trains of 7 pulses separated by 3 ms. When the HIP stimulation was combined with TA stimulation, the first TA stimulation pulse occurred with a 17 ms delay relative to the last pulse of the HIP stimulation train. In this way, the entire HIP stimulation train was completed well before the TA input pattern begun, which also resulted in that any lingering shock artifact from the HIP stimulation was prevented from impacting the response evoked by the TA input. This was done as we were not interested in the direct effects of HIP stimulation on the processing of the TA input, but rather the long lasting state change in the cortical network induced by HIP. Each of the 8 TA input patterns were repeated 50 times in isolation and 50 times in combination with the HIP stimulation (obtaining a total of 16 stimulation patterns). Only one of the 11 cells failed to reach 50 repetitions and instead had 44 repetitions of all stimulation patterns. In some cases (N = 7 recordings), where the neuron recording continued to be of a high quality, additional rounds of the protocol were commenced (for a total of 61–150 repetitions). All stimulation patterns were delivered in a pseudo-random order (i.e. a random order that was re-used across each experiment) separated by a randomized interval of about 1.8 s. In the cases where the first protocol was completed, we also applied the HIP stimulation in isolation (100 repetitions separated by 1.2 s) (N = 7 recordings).

#### Histological process, clearing technique

##### Cresyl violet acetate staining

For postmortem assessment of the position of the stimulation electrode, a subset of the animals were perfused with 4% PFA. At the end of each experiment, an i.v. overdose of ketamine/xylazine followed by transcardial perfusion with volume of ∼150 mL room temperature saline (0.9% NaCl in distilled water) and then 300 mL of 4% paraformaldehyde (PFA) in 0.1 M phosphate buffer (pH 7.4), which was kept cold on the ice. The extracted brain post fixated in 4% PFA for 48h and then cryoprotected by immersion in a 25% sucrose solution. After obtaining the equilibrium, the brain was frozen on dry ice and kept in the freezer. Then, the 25 μm thick sections (cryostat, Microm GmbH, HM 560, Germany) mounted on glass (Super Frost plus slides, Mänzel-Gläser, Germany) were kept at −25 ^⸰^C in the freezer. For the staining process, mounted sections were immersed in ethanol/chloroform (1:1) overnight at room temperature (RT). In next day, the process followed by rehydration in ethanol (95%), rinsed in distilled water (2 min/step), stained in Cresyl violet (Life Science product & service company; 0.1% in 0.3% acetic acid in dH_2_O; 5 min), rinsed in distilled water until sections became clear of extra dye, followed by dehydration in ethanol 95% (2 times 2 min), and clearing in xylene (2 × 100%, 5 min). At the end, the coverslip was placed on the stained sections using the DPX mounting media (Fluka, Germany). Slides were examined under light microscopy and images were obtained using DS-Ri1 digital camera (Nikon Instruments, Japan) mounted on a Nikon Eclipse 80i microscope.

#### Immunolabeling and iDISCO clearing technique for light sheet microscopy

##### Sample pretreatment with methanol

For another subset of the animals, the brains were prepared for light sheet microscopy to visualize the positions of the SI recording electrode and the HIP stimulation electrode. We used a standard iDISCO protocol (idisco.info) for PFA fixated frozen brain sample (one sample/one hemisphere). iDISCO is a method recognized for its ability in visualizing voluminous structures, therefore, the cellular profiles could be immunolabeled while retaining their morphological and molecular properties.^43^ In short, to de-freeze samples, they were washed in PBS (2x, 30 min each). Then, they went through a dehydration procedure, in different series of methanol/PBS dilutions: 20%, 40%, 60%, 80%, and in methanol 100% (2x)1 h/step. Then, they were incubated overnight in 66% DCM/33% methanol, RT with slight shaking (60 RPM) using the Ultra Rocker (Bio-Rad Laboratories, Sweden) in100% methanol (2x, 30 min each), then remained 1h at 4 ^⸰^C. Samples were then bleached in chilled fresh 5% H2O2 in methanol (1 vol 30% H2O2 to 5 volumes of methanol, ice-cold) over the night at 4 ^⸰^C. Next day, the brain was rehydrated with methanol/PBS series dilutions: 80%, 60%, 40%, 20%, PBS, 1 h/step, RT.

##### Immunolabeling protocol

Neurobiotin (Vector Laboratories, SP-1120) was included in the *in vivo* patch-clamp recording electrode solution (1.5% dilution). We also coated the HIP stimulation electrode in Neurobiotin for assessing its location within the brain. To increase the chances of detecting the tracer in the light sheet microscopy, we used a primary antibody against neurobiotin. Hence, bleached samples were incubated in the permeabilization solution buffer (PTx.2/glycine/DMSO) at 37 ^⸰^C for 5 days (d), then blocked in PTx.2/DMSO/Donkey Serum (Jackson laboratories) at 37 ^⸰^C for 5 days. Then they were incubated with the primary antibody (neurobiotin) (1:1000) in PTwH (PBS/0.2% Tween 20 with 10 μg/mL heparin)/5% DMSO/3% Donkey Serum for 9 days. Then, they were washed with PTwH for 10, 15, 20 min, 1 h, and 4–5 times more until the next day, followed by a 10 days incubation with the secondary antibody, streptavidin Alexa Flour-568 conjugated (1:500) (ThermoFisher, Catalog number: S11226) in PTwH/3% Donkey Serum at 37 ^⸰^C. Samples were finally washed in PTwH for 10, 15, 20 min, 1 h and then 4–5 times before the clearing step the next day. For all these steps samples were under slight shaking condition.

##### Tissue clearing

Immunolabeled samples were cleared following iDISCO method (idisco.info). Samples were dehydrated in methanol/PBS series: 20%, 40%, 60%, 80%, 1h each, twice with 100% methanol 1h each at RT and left over the night. Next day, they were incubated in 66% DCM/33% methanol, 3 h, RT, shaking. Then, incubated in 100% DCM (Sigma 270997-12X100ML), 15 min twice, shaking. In the final step they were incubated in Ethyl Cinemate (Sigma-Aldrich, SKU: 112372-100G) until clear and stored in RT until imaging with light sheet microscopy.

##### Light-sheet microscopy

The cleared hemisphere imaged with an Ultra Microscope II (LaVision Biotec) carrying an sCMOS camera (Andor *Neo* model 5.5-CL3), which the objective lens 1.3X (LaVision LVMI-Fluor 1.3x70.08 MI Plan) was used and 600/30 emission for Alexa Fluor 568. Stacks were made by overlapping up to 10% and stitched via Arivis Vision 4D 3.01 software.

### Quantification and statistical analysis

#### Post-processing of recorded neuronal data

The raw neuronal membrane voltage recordings were post-processed by first defining a stable baseline voltage by applying a least-square linear detrending to 10 s long segments. The recordings were subsequently low pass filtered using a rolling average of 100 bins equating to a boxcar window with a duration of 1 ms. In recordings where occasional spikes appeared, the onset of these spikes were found using a spike shape template and a recursive fitting algorithm.^44^ Stimulation artifacts and any spurious action potentials were removed by linear blanking. Finally, the recordings were down sampled to 1 kHz. Overall, the software used in the below analysis was NumPy and SciPy, standard Python libraries in data science.

#### Definition of evoked responses

The time window included in the analysis started at 5 ms after the onset of the TA stimulation (i.e., for both TA and HippTA stimulation patterns, corresponding to the conduction time from the skin stimulation to the earliest possible arrival of synaptic responses in the neocortex) and ended 400 ms later (some TA patterns lasted almost 350 ms, and responses could sometimes be detected at least 50 ms after the last stimulation pulse).

#### Clustering method

We used the same method to cluster the responses as we used in a previous publication (Etemadi 2022). This method identifies clusters of similar responses, with respect to their time-voltage curves, evoked by the same stimulation pattern, in an unsupervised manner. Hence, the algorithm was designed to identify clusters where the ‘members’ of each group were internally similar, while also being distinct relative to the other responses evoked by the same stimulation pattern. Furthermore, the algorithm had to be able to automatically determine the number of clusters that could exist, since this could not be known *a priori.*

First, each response was *Z* score normalized, i.e., the response mean over the whole time period was subtracted from each response and then the remainder of each response was divided by the standard deviation. Secondly, a distance matrix was calculated for the whole set of responses. The distance matrix was populated by calculating the M number of Principal Components (PC) explaining 95% of the variance for N-1 responses. All responses were then fitted to the PCs using the least square method. The resulting PC coefficients were used to position the responses in the M-dimensional PC space. Within this M-dimensional space, the Euclidean distances between a reference response and the rest of responses were calculated. The distances between the reference response and each of the other responses were then put in a matrix and the procedure was repeated until each response had been the reference. Finally, the distances were normalized to a 0–1 range.

From the Euclidean distance calculations above, a linkage matrix was calculated using the hierarchical clustering approach of Ward’s minimum variance method.^45^ This method sequentially finds the two closest neighboring responses with respect to their Euclidean distance. Then it finds the pair of the second closest neighboring responses and so on until there are no responses left. However, a response may also be closer to a center point between a pair of responses than it is to other responses, in which case the distance to that center point is assigned to the response. This distance is called the cophenetic distance. Sometimes, the shortest distance that can be found is between two such center points. All these distances are used to create the hierarchical structure of responses, where the distances between all such pairs can be compared.

The next step is to identify the cophenetic distance at which the identified clusters are objectively the most separable. The total cophenetic distance of a hierarchy was divided into 100 steps. Each level of cophenetic distance was used as a threshold, which would separate clusters from each other (i.e. a cluster of responses with a cophenetic distance larger than the threshold was considered a unique cluster), whereby an assignment of a cluster id was made. At this point we could perform data shuffling if needed. Data shuffling was achieved by shuffling the leaf position of each response in the hierarchy (the shuffling was performed using the NumPy shuffle method), therefore disrupting the relationship between cluster and response.

We next calculated the separability of the clusters by using a decoding analysis (described in detail in ‘Evaluation of cluster separability using decoding analysis’ below). The decoding analysis yielded, for each clustering threshold, a measure of the separability of the clusters as the F1-score. With the F1-score, we could apply Gap statistics to identify the cophenetic distance providing the largest cluster separation relatively to the shuffled data. Meaning that for each of the 100 cophenetic distances considered, we obtained an F1 score for the non-shuffled data and another F1 score for the shuffled data, subtracting the F1-score of the shuffled data from the F1-score of the actual data yielded the difference also known as the Gap Statistic.^46^ The calculation of the Gap Statistic was repeated three times per clustering threshold and the mean value was reported. Since the specific value of the chosen cophenetic distance defines the number of clusters to be considered, we could identify continuous stretches of cophenetic distances where the number of clusters, and the clusters themselves, remained unchanged. Over each of these continuous stretches we calculated a mean Gap Statistic. The middle value of cophenetic distance of the stretch with the maximum Gap statistic was used to define the cophenetic distance at which the clustering was objectively the most distinct. The clustering obtained at this value was then used for the remainder of the analysis and displays.

While following the above principles, there were some special situations that could arise. A cluster was assumed to be valid only if it had five or more members. All non-valid clusters were put in the same undefined cluster (‘unclassified’ responses). In addition, if the situation arose that one of the clusters consisted of only one member this response and cluster was excluded from all subsequent analysis and displays.

#### Evaluation of cluster separability using decoding analysis

We used the same general decoding algorithm to determine the specificity of the response clusters as in several previous publications,^6^^,^^17^^,^^18^^,^^29^ including analysis of intracellular recording data with multiple response states to each tactile input pattern.^13^

The responses and their respective label were split into a training- and a test set (80%/20% split) stratified by cluster label frequency. The training set was used to train a PCA model explaining 95% of the variance, and the coefficients obtained by fitting the responses with the least-square-method to the principal components were calculated for both the training set and the test set. Finally, a kNN-classification was performed with the training set coefficients as training data, and the test set coefficients as test data. This decoding algorithm was performed 30 times, each time with a new split of train and test data. The F1-score was calculated from the average classification result across the 30 repetitions.

The decoding analysis was also repeated with the randomly shuffled cluster labels (as described in Clustering method), reported as the “shuffled” context. This was done to establish the actual chance level of the data, as opposed to the theoretical chance decoding level (which equals 1 divided by the number of clusters).

## Data Availability

• The original contributions and raw data are deposited at figshare, https://doi.org/10.6084/m9.figshare.22758686.v1 • Further information about the analyzed data discussed in this paper will be provided by the lead contact upon demand. Code to perform the analysis will be provided by the lead contact on demand.
