# Supplementary material for: Hippocampal output profoundly impacts the interpretation of tactile input patterns in SI cortical neurons
Source: iScience. 2023 May 13;26(6):106885. doi: 10.1016/j.isci.2023.106885 (PMC10227419; doi:10.1016/j.isci.2023.106885)
Supplement: Document S1. Figure S1 [file mmc1.pdf]

iScience, Volume *26*

Supplemental information

Hippocampal output profoundly impacts the interpretation of tactile input patterns in SI cortical neurons

Leila Etemadi, Jonas M.D. Enander, and Henrik Jörntell

Supplementary Figure

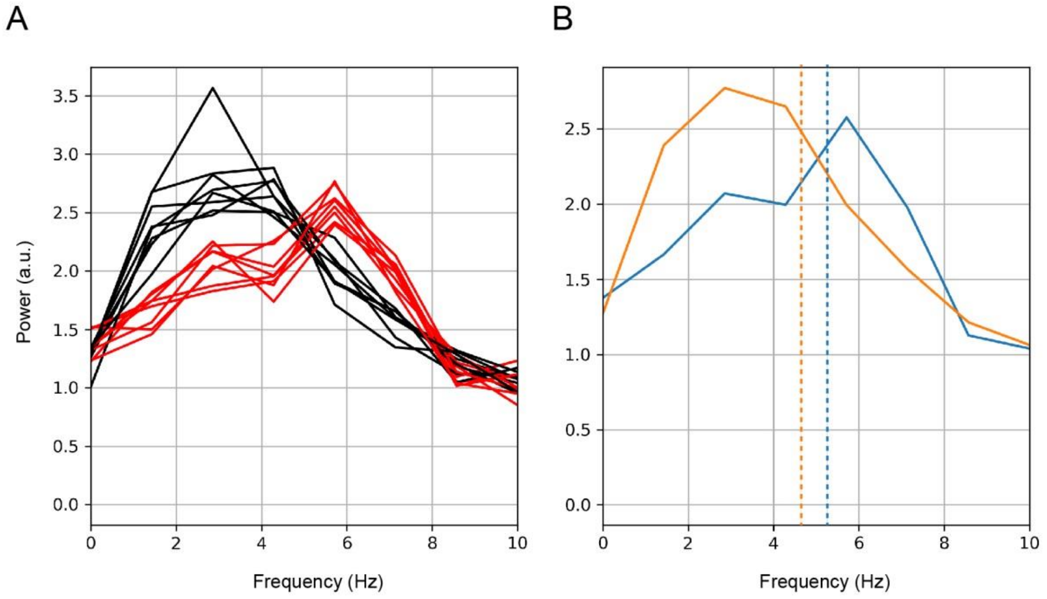

Supplementary Figure 1, Related to Figure 1F. Upward frequency shift induced by HIP stimulation. (A) Frequency-power curves for 8 TA inputs (black traces) and 8 HIPTA inputs (red traces) from one cell. (B) Average curves for TA (orange) and HIPTA (blue) inputs. Data were obtained from 700 ms raw data sweeps, 100 per input pattern. Each curve (A) represents the average frequency-power distribution after Fourier transform (1.45 Hz resolution) of each raw sweep. We then compared the average center of gravity (showed with dashed lines for the example cell in B) of the frequency power distributions for the average TA and the average HIPTA curve for each cell. A paired Wilcoxon signed rank test indicated a significant upward shift in the center of gravity caused by the HIP condition across the population of cells (N=9, alpha level $P < 0.05$). Notably, 2 of these 9 cells failed to show such an upward shift, whereas it was consistent in the other 7 cells.
